# Building a UAV Based System to Acquire High Spatial Resolution Thermal Imagery for Energy Balance Modelling

**DOI:** 10.3390/s22093251

**Published:** 2022-04-23

**Authors:** Krisztina Pintér, Zoltán Nagy

**Affiliations:** 1 MTA-MATE Agroecology Research Group, Hungarian University for Agriculture and Life Sciences, Páter K. u. 1., H-2100 Gödöllő, Hungary; 2 Department of Plant Physiology and Plant Ecology, Institute of Agronomy, Hungarian University for Agriculture and Life Sciences, Páter K. u. 1., H-2100 Gödöllő, Hungary

**Keywords:** UAV, RGB and thermal imagery, evapotranspiration, TSEB, eddy covariance

## Abstract

High spatial resolution and geolocation accuracy canopy evapotranspiration (ET) maps are well suited tools for evaluation of small plot field trials. While creating such a map by use of an energy balance model is routinely performed, the acquisition of the necessary imagery at a suitable quality is still challenging. An UAV based thermal/RGB integrated imaging system was built using the RaspberryPi (RPi) microcomputer as a central unit. The imagery served as input to the two-source energy balance model pyTSEB to derive the ET map. The setup’s flexibility and modularity are based on the multiple interfaces provided by the RPi and the software development kit (SDK) provided for the thermal camera. The SDK was installed on the RPi and used to trigger cameras, retrieve and store images and geolocation information from an onboard GNSS rover for PPK processing. The system allows acquisition of 8 cm spatial resolution thermal imagery from a 60 m height of flight and less than 7 cm geolocation accuracy of the mosaicked RGB imagery. Modelled latent heat flux data have been validated against latent heat fluxes measured by eddy covariance stations at two locations with RMSE of 75 W/m^2^ over a two-year study period.

## 1. Introduction

Information on the spatial distribution of evapotranspiration (ET) is becoming increasingly important in agriculture and in water management with the higher frequency of droughts in many areas of the world [1]. Satellite-based products/applications for ET mapping [2] are already commonly available for this purpose. However, data products such as MODIS Evapotranspiration (500 m spatial resolution) or Landsat Provisional Actual Evapotranspiration (30 m spatial resolution) or Sen-ET produced ET maps by applying thermal sharpening procedure (20 m spatial resolution [2]) have still too large pixel sizes regarding spatial accuracy for studies conducted on, e.g., evaluating small scale field plot experiments or correlating the modelled latent heat flux/evapotranspiration data to plant ecophysiological data (for example leaf/stand gas exchange or leaf area index) possibly measured on the ground. Other factors such as cloudiness or time of satellite overpass may also limit the periods when satellite imagery can be used. The effect of pixel size on ET model performance was also shown to be significant when modelling systems consisting of different plant functional types [3], ET was accurately simulated when the pixel scale was suitably small (<5 m) to robustly discriminate between grass and tree pixels, and model uncertainty drastically increased at a spatial resolution greater than 10 m [3]. In Nassar et al. [4] model-measurement differences were also shown to be increasing in a vineyard (e.g., no occurrence of different plant functional types) when decreasing the spatial resolution. Moreover, Kustas et al. [5] pointed out that when there is a significant discontinuity in surface conditions, the subpixel variability in energy fluxes will likely cause unacceptable errors in the ET prediction. These findings support the need for high spatial resolution ET mapping (e.g., from drones) to enhance the accuracy. The trade-offs between the expectations towards efficient resource use, high spatial resolution, and accuracy of thermal images led to consider solutions where main parts (basically the thermal and the RGB/multispectral cameras) can be flexibly and independently used in UAV based systems.

With the development of UAVs and imaging sensors attached to them [6,7] it became possible to collect information from suitably large areas and thereby to provide valuable field-scale information also for precision agriculture [8] applications. Along with this trend, there was an increased interest in UAV based ET estimation in the scientific community. Niu et al., in [9], summarized the studies published on this topic in the last couple of years and found that the UAV based remote sensing can be a more flexible solution in precision agriculture applications then satellite based. UAV based ET mapping was applied for various vegetation types, from grasslands through barley fields to olive orchards and vineyards (Table 1). Table 1 is based on Niu et al.’s [9] summary, but it is complemented with a few more recent studies and the ground resolution of the different cameras. It must be noted that the type of UAVs and imaging sensors vary in a wide range in the early studies, while in the most recent manuscripts the DJI drones and MicaSense cameras are dominating.

Applications using two or more different proximal sensing systems on one UAV platform on the same occasion are frequent and the expectations toward such integrated systems—considering both the higher number of applications in a single flight and the geolocation accuracy—are increasing. Proximal sensing of temperature in parallel with multispectral RGB sensing is an example of that, but there are other ones including (RGB + radar, multispectral + non-imaging spectrometers). While the popularity of the off-the-shelf drones is understandably high (no need to bother with solutions/applications often difficult and time-consuming to develop), the price of these systems and flexibility/quality expectations against the applications may require investing some work for integrating different systems into one functional unit. High spatial resolution (higher than achievable by off-the-shelf solutions) of thermal cameras can be such a reason for using a thermal camera together with an RGB camera to apply the imagery as input to an energy balance model (e.g., the pyTSEB model, [13,14]) to create evapotranspiration maps. Emerging needs for better spatial resolution of thermal cameras are also shown for example by the observable trends of the industry to replace existing solutions of multispectral/thermal applications with ones of higher thermal resolution. Higher spatial (geolocation) accuracy is another issue when the goal is plant phenotyping in a small-scale field plot experiment setup. In such cases, commonly achievable 1–3 m accuracy as provided by onboard GNSS (Global Navigation Satellite Systems) rovers is not satisfactory. Use of ground control points (GCP) while overcomes this problem, is a tedious and time-consuming procedure, takes time from the short periods available for image acquisitions when other constraints (to have steady light conditions during flight, the time necessary for ground control measurements, the time necessary to put out and collect GCPs) should also be considered and met. Therefore, the use of RTK/PPK systems is necessary though the costs of the two approaches are highly different (RTK in most cases is more complex and therefore a more expensive solution).

The base of solutions flexible enough to incorporate a thermal camera or a chosen GNSS rover is frequently one of the Raspberry Pi microcomputers (RPi) models [22]. RPis are widely used in different agricultural applications [23,24] where they are preferred for the availability of many different interfaces/ports (GPIO, UART, etc.) provided and for accessible software development kits (SDK) that can be installed on this platform. Integrated applications of component devices can be developed by (slightly) modifying the provided SDKs. It is to be noted here that, SDKs have usually passed a quality assurance process and many of the issues requiring a long time by an experienced engineer are already solved in them so that, limited programming experience may satisfy the needs of the development of a custom integrated application.

The development of such an integrated application was aimed to produce a UAV-based thermal and RGB image acquiring system that collects high spatial resolution thermal data while also enabling the user to high spatial location accuracy by applying PPK processing. The resulting imagery served as part of the input to model evapotranspiration by using the TSEB (pyTSEB) energy balance model [15]. Modelled evapotranspiration/latent heat values from the eddy covariance (EC) footprint area were compared to the measured fluxes. Validation was carried out by EC data and modelled maps of ET using data from several flights. The application can be developed further by, e.g., including further miniature sensors (a spectrometer, or temperature/humidity/other scalars sensor) to be controlled from the same RPi or using possibilities provided by the onboard SDK. Knowledge of the spatial distribution of transpiration and evaporation is expected to have importance in evaluating small scale field plot experiments in field-scale plant phenotyping trials and generally in applications where subpixel heterogeneity may be expected to occur. Conforming to expectations towards producing ET maps of suitably large spatial coverage, resolution and accuracy at reasonable efforts may require considering use of independent parts that can flexibly used in a common UAV based system.

This study shows an example of conforming to the complex constraints of acquiring high spatial resolution thermal imagery of reasonably high spatial resolution and accuracy, and use this data in a spatial modelling context to produce evapotranspiration maps. These maps are good candidates to be used in evaluation of field scale trials on plant production, irrigation, plant phenotyping trials as ET is frequently dominated by transpiration, a single variable that is mechanistically linked to gross primary production through stomatal action. The performance of the procedure was evaluated by validation of the produced ET models against ET as measured by eddy covariance technique on a diverse dry grassland and on an arable land.

## 2. Materials and Methods

We aimed to set up a system onto the board of a UAV that can take RGB images and thermal snapshots while registering the geographical coordinates of the camera events and subsequently produce georeferenced RGB and thermal mosaics from the gained dataset to be used as inputs of a model estimating evapotranspiration. Our setup’s hardware consists of a commercially available digital camera (Canon Ixus 160/Sony a6000) a thermal camera (OPTRIS PI 400/640, Optris GmbH, Berlin, Germany), a GNSS receiver chip (M+/M2, EMLID Ltd., Hong Kong, China), while the main, controlling and data storing device, is a Raspberry PI 3B single-board microcomputer (Raspberry Ltd., Cambridge, UK). The control of the whole system was based on The Optris Software Development Kit (Evocortex GmbH, Nürnberg, Germany) installed on the RPi.

### 2.1. UAV Setup

**Figure 1:**
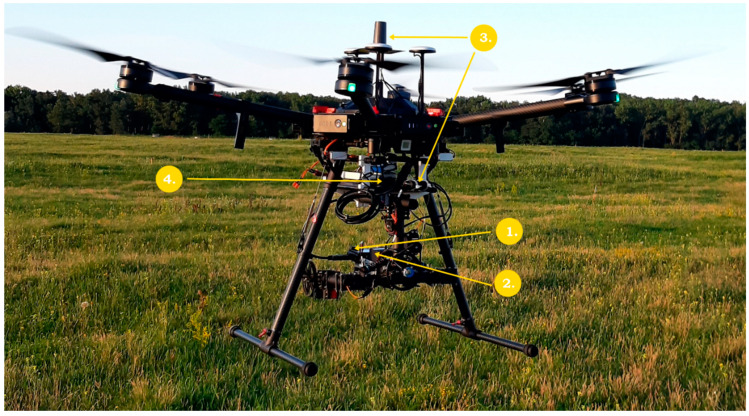
Picture of the DJI M600 UAV and the equipment mounted on it. The numbers and the arrows indicate the different parts of the imaging system. 1: Sony a6000 RGB camera, 2: Optris PI 640 thermal camera, 3: Emlid Reach M2 GNSS receiver and its antenna, 4: Raspberry 3B single-board microcomputer.

The multicopter used for surveys is a DJI Matrice 600 Pro hexacopter equipped with a 2D carbon frame gimbal providing nadir view positions for the thermal and RGB cameras installed on the gimbal. The cameras have been fixed by using their ordinary main fixing screws (Figure 1). Plastic structures were designed, and 3D printed to hold the gimbal and a plastic sheet as a platform (Figure 1) for carrying the Raspberry Pi 3B, the M+/M2 GNSS (EMLID) receiver, the LiPo batteries (11.4 V, 3S) to supply current for the devices. All the cameras/devices (including the gimbal) obtained electric current supply independently from the drone’s power supply.

### 2.2. RGB and Thermal Cameras

In the initial setup, a Canon Ixus 160 digital camera was chosen considering its lightweight and high resolution (20 Mpix) and because of the possibility to be triggered through its mini-USB port. To establish the trigger a homemade printed circuit (PCB) had to be prepared (see details of the triggering later). After the first season’s flights, the Canon camera was replaced by a Sony a6000 (23.4 MP). On one hand, the Sony a6000 is a superior camera as compared to the Canon Ixus 160 (Table 2), on the other a direct connection between the camera and the GNSS receiver could have been established through after the hot shoe cable becoming available on the market. The direct connection provides better spatial accuracy when using PPK (see sections on georeferencing and software control). Thermal images were taken by an Optris PI 400 thermal camera (replaced by an Optris PI 640 after the first year), with an optical resolution of 382 × 288 pixels (640 × 480) and spectral range of 8–14 µm. The camera connects via its USB port to the Raspberry Pi 3B.

### 2.3. Software Development for Control of the Devices and Data Storage

The IRImager Direct Software Development Kit (SDK, Copyright (c) 2016–2017, Optris GmbH/Evocortex GmbH, All rights reserved.) is published in C++ language. The SDK provides the users with the possibility to control (select operation mode, acquire image features, trigger etc.) the camera and save pictures from the data stream. The SDK was installed on the RPi running a Linux (Ubuntu Mate 16.04) operating system according to the instructions on the home page of the SDK (http://documentation.evocortex.com/libirimager2/html/index.html accessed on 25 August 2017).

An example script (*irimagerShow.cpp*) provided as part of the SDK was the base of our modified script. The main parts of the original script were kept, however parts of real-time data visualization were removed. The retained parts of the script performed the following tasks: establishing the link between the thermal camera and the RPi, initializing the operation of the thermal camera, performing calibration during operation and error handling. Modification of the code includes establishing RS232 communication with the GNSS receiver (Appendix A Figure A2), obtaining and storing NMEA sentences, taking a thermal snapshot from the stream at regular intervals, providing a trigger signal to the RGB camera, and writing necessary (GPS and temperature) information into a .txt file (Appendix A Figure A3). We have also put in some code lines to receive notifications if the communication between the RPi and the GNSS receiver was proper and if the M+/M2 was obtaining a proper quality (fixed) signal.

#### Control of the OPTRIS Thermal Imager and RGB Camera

**Figure A1:**
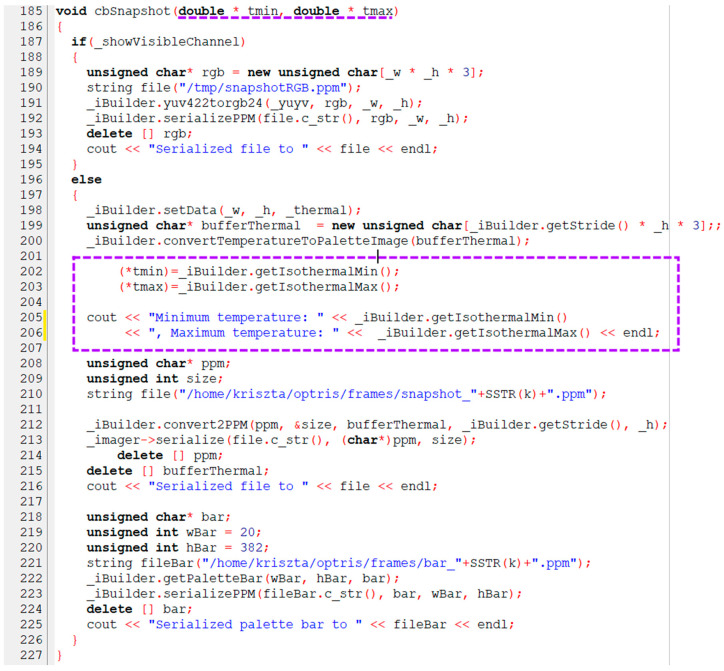
Controlling script of the thermal imaging system, part 1: modification of the *cbSnapshot()* function. The purple dashed frame and a purple line highlights the inserted parts.

By default, the output of the Optis PI400/640 thermal camera is a video stream from which snapshots (images/frames) can be saved by the *cbSnapshot()* function of the SDK. The *cbSnapshot()* function was complemented with further SDK functions (*_iBuilder.getIsothermalMin()*; *_iBuilder.getIsothermalMax()*) to obtain the minimum and maximum temperatures for the actual frames, respectively (Appendix A Figure A1). These data were required during the rescaling process (described in Section 2.6.2) before mosaicking. Saving the thermal images as .ppm (Portable Pixmap Format) files to the RPi’s storage is also controlled by the *cbSnapshot ()* function.

**Figure A2:**
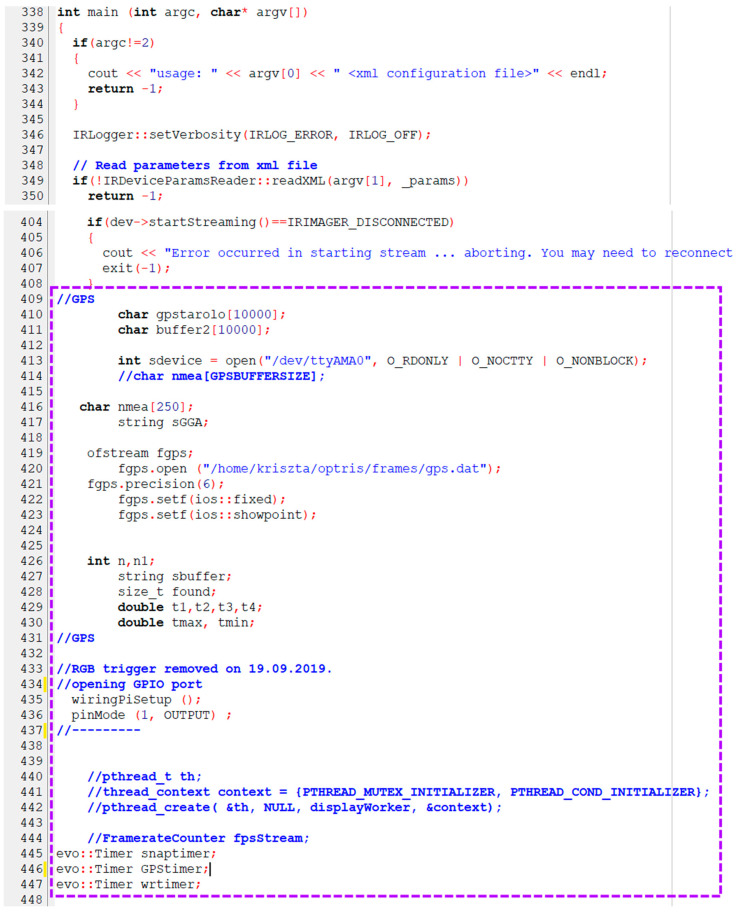
Controlling script of the thermal imaging system, part 2: Opening of the serial port and a text file output, reading the GNSS information, initialization of the GPIO 14 port and of built-in timers, declaration of the additional variables used in the script. The purple dashed frame highlights the inserted parts.

Initialization of the serial communication with the GNSS receiver, opening of the. txt file storing GPS and temperature information was also added to the script (Appendix A Figure A2). The GPIO 14 port triggering the digital camera was configured as output by the *wiringPiSetup()* and *pinMode()* commands of the WiringPi library (http://wiringpi.com accessed on 29 November 2017).

**Figure A3:**
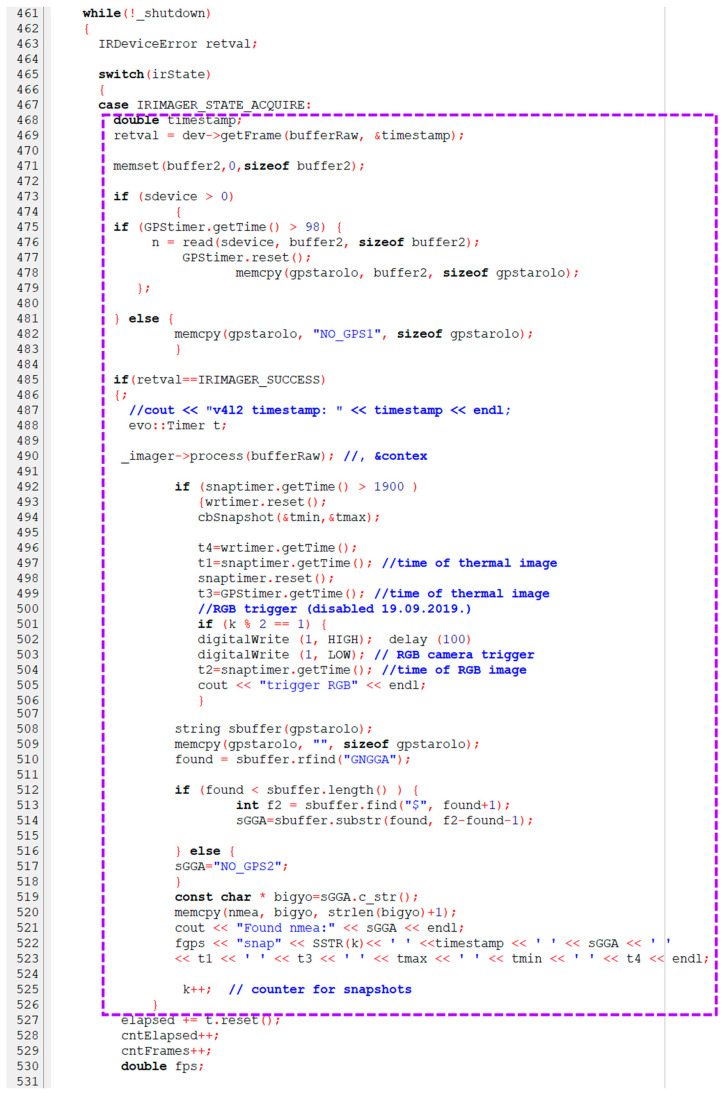
Controlling script of the thermal imaging system, part 3: Reading of the GNSS information from the serial port, initiating the thermal snapshot, triggering the RGB camera, and writing the necessary information into a text file The purple dashed frame highlights the inserted parts.

A conditional (“while”) loop (called in every 1900 ms) was inserted into the inner part of the script (Appendix A Figure A3), with all commands necessary to control the parts of the system and save collected images and information included in this loop.

The first of these commands was the one calling the *cbSnapshot()* function initiating the retrieval of the thermal frames.

After the *cbSnaphop()* function was completed, RPi GPIO 14 (UART TX) was set to high for 100 ms by the *digitalWrite()* command of the WiringPi library. This command triggered the Canon Ixus 160 camera via the home-made PCB connected to GND, 5V, GPIO14 and 15 ports of the RPi.

Afterwards, the serial port was polled for GPS data availability. The serial number of the actual snapshot, the corresponding maximum and minimum temperature and the GPS data were saved to a text file.

**Figure 2:**
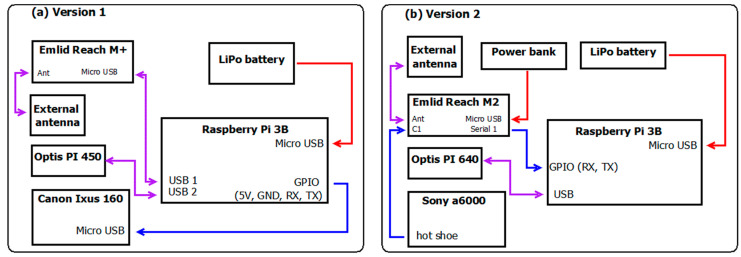
Schematics of the thermal camera system. The arrowheads indicate the direction of communication. Red arrows indicate power supply, blue arrows indicate data stream, purple ones indicate power supply and data stream together. Version 1 (**a**) was used in the first year of the study. After changing some parts of the system, Version 2 (**b**) was used in the 2nd year.

The camera controlling script slightly changed when the Canon camera was replaced with a Sony A6000 in the second half of the first study year. The Sony A6000 camera was time interval triggered from the menu and was equipped with a hot shoe connector triggering a time stamp store on the Emlid Reach GNSS receiver (Figure 2). Using this setup meant to lose common control (triggering and taking a frame, respectively) of the thermal and the RGB camera from the same code. Geolocation accuracy of the RGB image, however, improved by using the standardized procedure applied on (hot shoe-triggered) timestamps stored in a text file on the M2 GNSS receiver (https://emlid.com accessed on 19 March 2020).

### 2.4. Equipment for Georeferencing

For precise georeferencing GPS data obtained from the autopilot of the UAV is usually not enough as the accuracy of these data is typically in the order of a couple of meters. A higher (even centimeter) level of geolocation accuracy can be achieved by employing Real-Time or Post-Processed Kinematics (RTK or PPK) methods. Both methods require the operation of a base station collecting raw GNSS information or data from an NTRIP server in addition to the rover’s (on the UAV platform) GNSS data. The RTK technique requires a constant good quality airlink connection between the rover and the base. However, in the case of the PPK method the constant connection is not needed and correction of the GNSS data collected during the flight is carried out by post-processing. We have used the PPK method in this study. Raw GNSS data were collected by a STONEX S8 GNSS receiver used as a base station during the flights. The location of the base station was measured prior to the flight with high accuracy by averaging coordinates collected during two minutes. During this measurement, an NTRIP connection to a nearby server supplying the RTCM3 correction signal was used. The base station was disconnected from the NTRIP server afterwards and was recording the GNSS data during the flight for PPK processing. Some details of the applied PPK process are described in Section 2.6.1.

An Emlid Reach M+ chip was used as the rover GNSS receiver onboard the UAV. The rover (the M+ chip) was set up by a mobile phone EMLID application to save the raw GNSS data at 5 Hz while streaming NMEA data through a USB connection to the Raspberry PI 3B. The M+ chip was replaced by an Emlid Reach M2 for faster start up and more stable operation. With M+ it might take to 20 min to have a fix signal with M2 it took 1–2 min and signal quality also improved significantly, after the exchange practically all the path was of Q1 quality (green on the application map in the RTKpost application). The Emlid M+ GNSS rover was receiving power supply from the RPi whereas the Emlid M2 from a separate USB power bank because the M2 requires larger power than available from the RPi USB. The data from M2 was sent using the separate serial connection between M2 and one of the RPI’s GPIO ports, while the micro USB port was used both for power supply and communication in the case of the M+. Setup of the GNSS receivers (GNSS networks used, recording frequency, storage, etc.) was carried out by using the mobile phone application provided by the supplier. The raw GNSS data of the rovers and later the stored coordinates of trigger events (NMEA sentences) were post-processed (PPK processing as described in the Section 2.6).

Ground Control Points (GCP) were installed and their locations were measured with high accuracy using the Stonex S8 GNSS receiver set up as a rover in RTK mode at both the cropland and the grassland sites. The GCPs were 16x16 cm wooden squares coated with thick aluminum foil so that they could be detected on the thermal images as well. The wooden squares were fixed atop poles of height providing visibility, e.g., 40 cm height on the grassland and 90 cm height on the cropland.

### 2.5. Study Sites

UAV based image acquisition campaigns were conducted in 2019 and 2020 on two sites (Table 3). One of them is a dry permanent grassland used as pasture in the Kiskunság National Park (19.6° N, 46.69° E, 104 m, Bugac, Hungary) the other is cropland (19.53° N, 47.66° E) near Kartal in the northern part of Hungary. The study sites are equipped with eddy covariance (EC) towers measuring the turbulent exchange between the vegetation and atmosphere. The measured turbulent fluxes include the sensible and the latent heat fluxes, used during the ET model validation process in the present study. The EC station was set up in 2002 at the grassland site and in 2017 at the cropland site.

The eddy covariance system consists of a CSAT3B Sonic Anemometer (Campbell Scientific Ltd., Logan, UT, USA) and an LI-7500 open path CO_2_/H_2_O infrared gas analyzer (LiCor Environmental, Lincoln, NE, USA) at both sites. Net radiation is measured by an NR Lite net radiometer (Campbell Scientific Ltd., Logan, UT, USA). A more detailed site description for the grassland and the setup of the eddy covariance systems valid at both sites have already been reported in [25,26]. The cropland site is on a productive soil of high clay content with millet and wheat sown in the two study years. The minimum fetch is about 100 m at this site, so the main devices of the EC system were installed at a variable height of 1.7–3.5 m above the ground.

### 2.6. Data Processing

#### 2.6.1. GNSS Data Processing

Raw base data saved by the STONEX S8 GNSS receiver was downloaded by the STONEX RTK Assistant (ver 1.62) application in RINEX format. Rover data from the Emlid Reach M+/M2 chips was downloaded by the ReachView (Emlid Ltd., Hong Kong, China) application.

The raw GNSS data collected from the base and rover modules were post-processed by the tools provided by Emlid Ltd. These tools are based on the RTKLIB Open Source Program Package and were optimized by Emlid Ltd. to be used with data collected with GNSS receivers manufactured by Emlid. The raw GNSS data was collected in .UBX format, and as a first step it was converted (rtkconv.exe) to .nav and .obs files. In the next step, these files together with the RINEX OBS and RINEX NAV files from the base station were processed by the rtkpost.exe application. The result of this step is a .pos file containing the coordinates of the route of the UAV in 5 Hz resolution (i.e., every 0.2 s). When the Sony A6000 digital camera was connected to the Emlid Reach receiver (rover) through its hot shoe then an additional output file was generated by the application (rtkpost.exe) containing the post-processed coordinates of the camera trigger events. The output files also contain the GNSS solution quality (fix, float, single) of the coordinates. As in the mosaicking software (Agisoft Metashape) the position accuracy information was expected in a term of distance (shift), the quality codes were converted based on our arbitrary choice as follows: fix: 0.5 m; float: 2 m; single: 10 m.

In the case of the thermal images, the GPS data saved as a text file saved on the Raspberry PI 3B could not be post-processed directly by the RTKLIB applications. Instead, Post-processed GNSS data was picked from the corresponding (5 Hz) PPK output dataset containing the whole route according to the timestamps of the thermal camera trigger events saved in the .txt file on the RPi. This selection was performed in an R environment.

#### 2.6.2. Thermal Image Processing

As the Optris PI 450/640 camera uses different temperature ranges at the different snapshots, a common temperature range for each flight had to be defined, and the images had to be re-scaled accordingly. This rescaling step is necessary before running the image mosaicking software to construct the map of surface temperatures.

Rescaling was performed in the R environment, thermal images were read with the *read.pnm()* function of the pixmap package as a first step in this procedure. This function returns a 3 channel (each 8 bits) RGB pixmap object for each thermal image. The 3 × 8 bit RGB values were converted to single 24 bit values by using a linear combination of the data in the RGB channel, as

(1) $$\left(256^{3}-1\right)R+\left(256^{2}-1\right)G+255B$$

**Figure 3:**
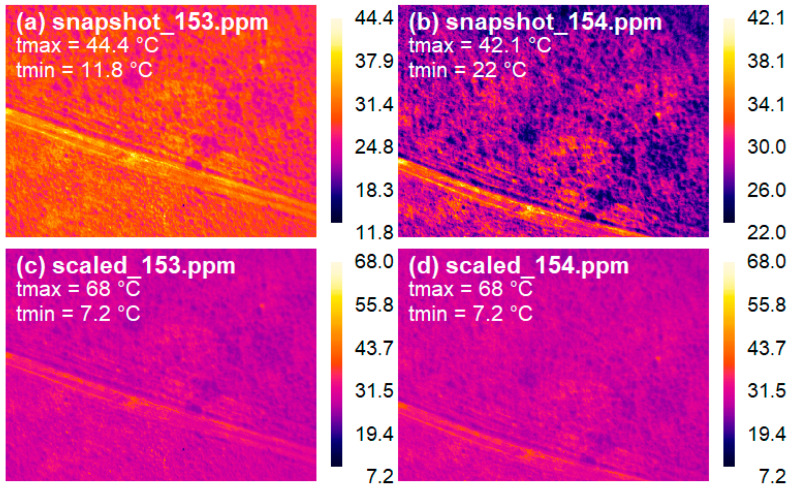
Illustration of the conversion process of thermal images. Raw thermal snapshots (**a**,**b**) are scaled to a common color (and temperature) range (**c**,**d**). The name of the file and the minimum and maximum temperatures of the images are shown in the upper left corner of the images.

This value was the input to calculate the pixel’s temperatures using the stored maximum and minimum temperatures of the given thermal image. Based on this single value an unambiguous attribution can be made between the pixel colors and temperature values. The colorbar saved with each snapshot (thermal image) contains 239 different colors, the brightest color (highest value) corresponding to the maximum temperature of the image while the darkest color (lowest value) to the minimum temperature. Similarly, the temperature range of each snapshot was divided into 239 values and the single 24 bit values were converted into temperature based on the attribution of the colors of the colorbar and the temperature range. In the next step, colors were attributed to the common (regarding the whole flight) temperature range. Based on the temperature calculated in the first step a new color based on the common colorbar was assigned to each pixel of a given snapshot then it was converted back to RGB values also based on Equation (1). The resulting images (Figure 3) were written with the *write.pnm()* function also from the pixmap package.

#### 2.6.3. Mosaicking of RGB and Thermal Images

The RGB images and the rescaled thermal images were mosaicked independently by the Metashape (Agisoft) software supplying the post-processed GNSS data in a separate file. The workflow was based on the tutorial published by Agisoft including—among others—camera optimalization and marking of GCPs. The latter step is supposed to enhance the spatial accuracy of the resulting maps. Four GCPs were left out of the procedure to make possible the evaluation of the spatial distortion of the geolocation. The outcome of the mosaicking process was two georeferenced 3-layered raster, one ordinary RGB raster, and one for the thermal images. In addition to the efforts made on enhancing the accuracy of GPS information by PPK, there was a slight misalignment between the two mosaics (RGB and thermal). As the GPS coordinates belonging to the RGB images were considered to be more accurate the thermal mosaic was aligned to the RGB in QGIS using the Georeferencer tool. The tool requires the marking of clearly discernible objects on the layer to be aligned and then obtaining their coordinates from the other layer. In this step, the same aluminum coated GCPs were used as in mosaicking. After the alignment, the thermal raster was converted to a 1-layer GeoTIFF containing temperature values with a procedure similar to the one described above rescaling the thermal snapshots to a single common temperature scale valid for a particular flight.

#### 2.6.4. Quantifying Spatial Error of the Mosaics

The accuracy of mosaicking and georeferencing was also attempted to be assessed. The idea was to measure the distance of known points at the RGB mosaics and in reality. In 2019, without GCPs being set, markers of a previously established grid were used. The markers were 2 cm by 2 cm wooden squares painted in white. They were placed 40 cm above the ground and were situated mostly in the central part of the RGB. In the next season, constant GCPs placed at the edge of the area of interest before the start of the 2020 season were used for this analysis. The location of the GCPs regarding all the RGB mosaics corresponding to the flights listed in Table 2 was marked and saved as a shapefile layer in QGIS. The distance between the location of the GCPs acquire from the RGBs and their measured coordinates were calculated by the *pointDistance()* function of the geosphere library in R.

#### 2.6.5. ET Modelling

The basic purpose of setting up the UAV system was to produce surface temperature maps and then turn them into evapotranspiration maps by the means of the pyTSEB model. The pyTSEB model is an energy balance model which calculates sensible heat flux from the temperature difference between the surface and the surrounding air, and then computes latent heat flux as the residual of the balance. The basics of the model are described in [15,27,28], in present work the Python version of the model (PyTSEB) was used, which was downloaded from is its GitHub source (https://github.com/hectornieto/pyTSEB accessed on 28 November 2019). The model is extensively used for evapotranspiration modelling both from UAV [12,14,17,18] and satellite-based thermal images [2,3].

In addition to its primary input—the surface temperature map/raster—there are other necessary inputs to run the pyTSEB model. These additional inputs include fractional cover (f_c_), green fraction (f_g_) and leaf area index (LAI) maps. Both, fractional cover and green fraction maps were produced based on the green layer of the RGB orthomosaic. Fractional cover of the grassland was considered as total (i.e., f_c_ = 1), and the green fraction was estimated, while in the case of the cropland all the vegetation was assumed to be green (f_g_ = 1), and the fractional cover was estimated from the green layer of the RGB. LAI maps were constructed using the regression between (ground) LAI estimations by a ceptometer and vegetation indexes (VI or VARI, whichever gave the higher R^2^) calculated from the RGB mosaic. The LAI and VI/VARI correlation were based on data from coinciding field survey activities when available. In the frame of these field surveys, LAI was measured at a spatial grid of 78 points with 30 m resolution. Without a coinciding survey, LAI was measured at 8 locations with highly different plant cover. In the latter case, after measurement, but prior to the flights, these locations were marked by 40 × 40 cm wooden squares which enabled us to extract the VI/VARI values without spatial bias.

As the last step of the data preparation, all the input rasters were resampled to the spatial resolution (~8 cm) of the thermal raster. Other surface parameters (vegetation type, vegetation structure parameters, micrometeorological variables) were compiled into a text file according to the format required by pyTSEB. The necessary micrometeorological data (air temperature, wind speed, pressure, vapor pressure, incoming solar radiation) was picked from the data measured at the eddy-covariance towers at corresponding the study site. The PyTSEB model was run from the conda command line. The main output of the pyTSEB model is a 4-layer GeoTiff image containing the net radiation (Rn), sensible heat (SHF) and latent heat flux (LHF) and ground heat flux (G), respectively.

#### 2.6.6. Validation of ET Modelling

Validation of the latent heat flux estimated by the pyTSEB model was performed by comparing it to the LHF measured by the eddy covariance technique. As EC based turbulent fluxes refer to a much smaller area than the area covered by the UAV flights, thus the area from which the EC fluxes originated had to be selected. This selection was performed based on the source area (footprint) analysis of the EC measurements. The footprint analysis is usually part of the flux calculation procedure, and its results are included in the output. The footprint calculation [29] in the EddyPro software outputs—among others—the main wind direction and the peak distance, i.e., the direction and distance from which the largest relative flux contribution is originating. The eddy flux footprint coinciding with the time of a given flight survey was used to find the area of interest on the ET map. We have used a rather simple representation of the footprint; it was considered as a 10 m diameter circle around the flux footprint peak. Pixels within the circle were averaged and then compared to the EC based LHF fluxes.

## 3. Results

### 3.1. Location Accuracy

The error of georeferencing (location accuracy of the RGB orthomosaics) is interpreted as the distance between the positions of the constant GCPs on the stitched RGB orthomosaic image and their actual positions (as measured by the STONEX S8 GNSS receiver in RTK mode) For the unavailability of markers (GCPs), in the first year, the analysis could be performed only for the Bugac. General lack of availability of constant GCPs is a common problem on croplands, as the cultivation operations require regularly remove GCPs.

**Figure 4:**
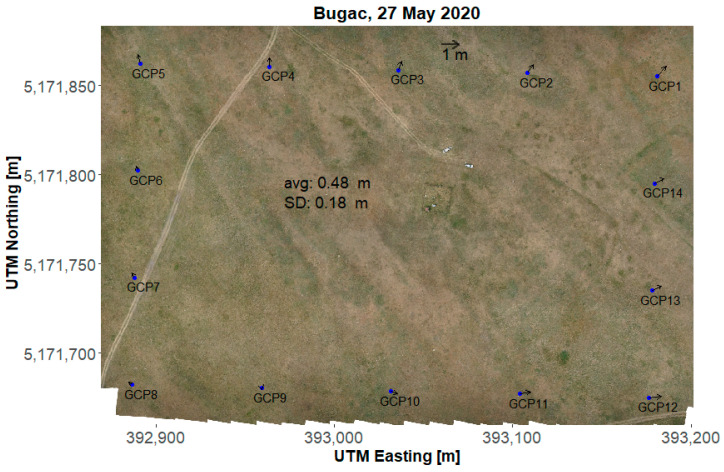
RGB orthomosaic from the Bugac (grassland) site taken on 27 May 2020. The GCPs on the RGB are highlighted with blue dots. The arrows point to the position where they should be according to their measured (STONEX S8) coordinates. The length of the arrays is multiplied by 10 for illustrative purposes, i.e., the 1 m arrow at the top of the image means 10 m at the orthomosaic.

**Figure 5:**
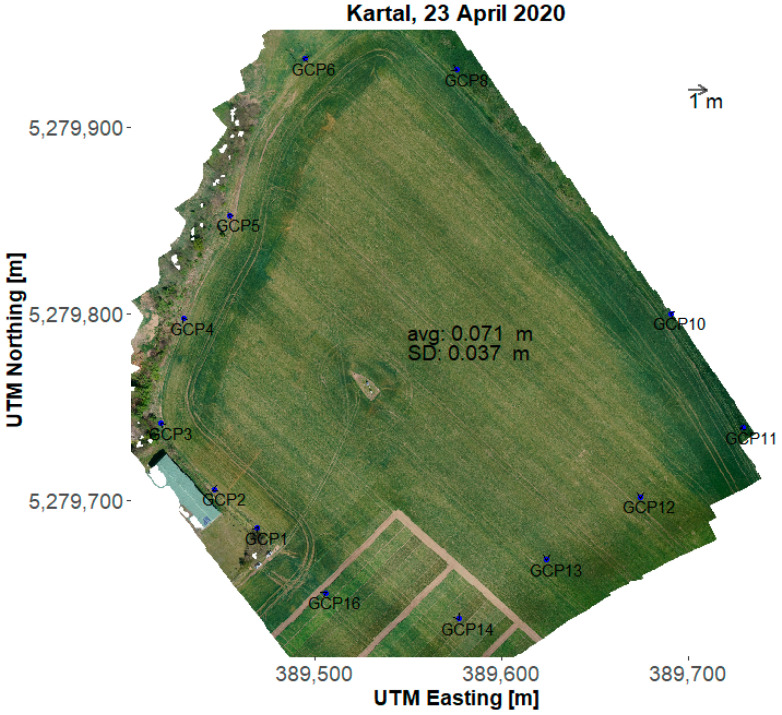
RGB orthomosaic from the Kartal (cropland) site taken on 23 April 2020. The GCPs on the RGB are highlighted with blue dots. The arrows point to the position where they should be according to their measured (STONEX S8) coordinates. The length of the arrays is multiplied by 10 for illustrative purposes, i.e., the arrow indicating 1 m length at the top of the image means 10 m at the orthomosaic.

The bias ranged from 0.22 m to 2.1 m, while the average was 0.825 m at the grassland in 2019 (data not shown). Examples of local spatial errors for 2020 are presented in Figure 4 and Figure 5 for the grassland and the cropland, respectively. The local spatial error of the RGB mosaic is represented by arrows pointing to the true position of the GCPs. The variable direction and magnitude of the error indicate that the error due to the inaccuracy of the position measurements and the subsequent mosaicking and georeferencing procedure was not constant in space. The high values of the spatial error in 2019 was turned out to be caused by communication delay. After upgrading the GNSS rover to a new model (Emlid Reach M2) the spatial bias decreased considerably Table 4 and Figure 4 and Figure 5. For the surveys in 2020, the spatial error at the grassland has a range of 0.2–0.5 m, while for the cropland site it is always less than 0.13 m. Average and standard deviation of the spatial error was calculated for the GCPs excluded from the mosaicking procedure (Table 4 lines avg (selected), sd (selected)). There is a considerable difference between the average spatial error for the two sites. After checking the raw data, it was found that the number of satellites when measuring the position of the base in RTK mode was considerably higher in the cropland (15–17) than it was in the grassland (8–9). Other accuracy measures also indicated lower accuracy in the grassland, e.g., position dilution of precision (PDOP) was between 1.65 and 1.99 in the cropland while it was between 2.2 and 2.6 for the grassland. Horizontal and vertical dilution of precision (HDOP and VDOP) was also generally higher in the grassland (HDOP: 0.9–1; VDOP: 2.0–2.3) than in the cropland (HDOP: 0.6–0.8; VDOP: 1.6–1.85). As these measures refer to the spread of the GPS satellites in the sky, improving the accuracy in this context is not straightforward.

### 3.2. Validation of the LHF Modelling

**Figure A4:**
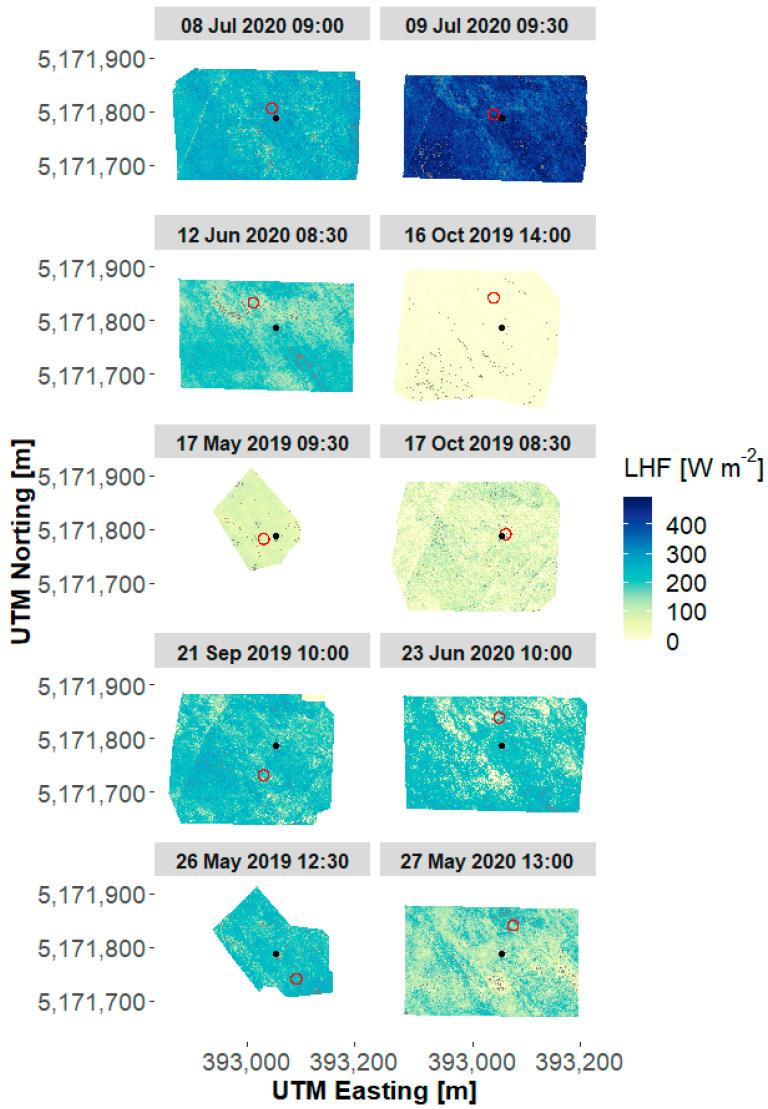
Latent heat flux maps of the grassland (Bugac) site in 2019 and 2020. The black dot indicates the position of the eddy-covariance tower, while he red circle indicates the footprint area from which the averages for validation were calculated.

**Figure A5:**
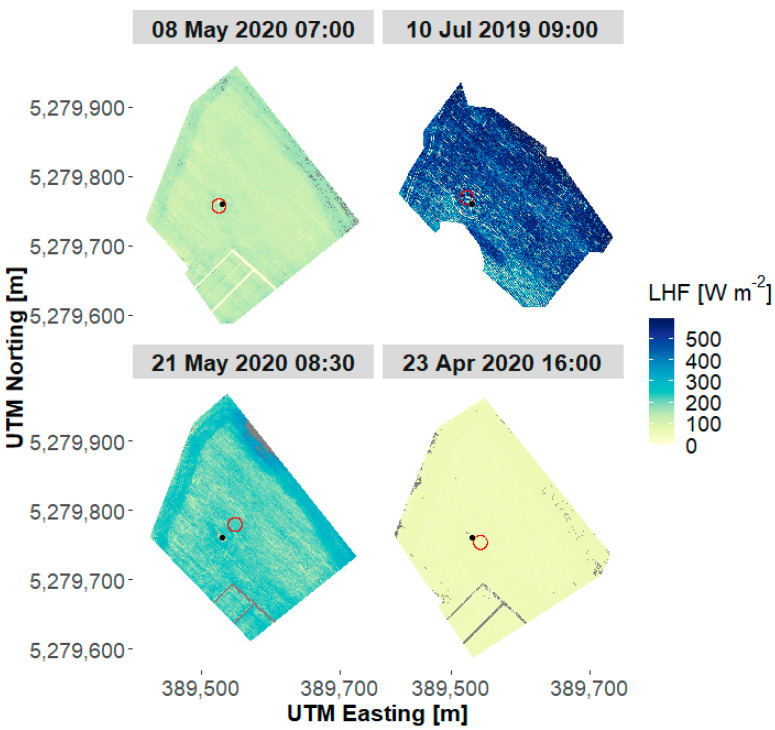
Latent heat flux maps of the cropland (Kartal) site in 2019 and 2020. The black dot indicates the position of the eddy-covariance tower, while the red circle indicates the footprint area from which the averages for validation were calculated.

There were 14 flights (10 at the grassland and 4 at the cropland) when all the necessary input raster were successfully produced and the pyTSEB model was run. The resulting LHF maps are presented in Appendix B Figure A4 (grassland) and Figure A5 (cropland). The footprint area taken into account when calculating the spatial averages for validation is indicated by circles

**Figure 6:**
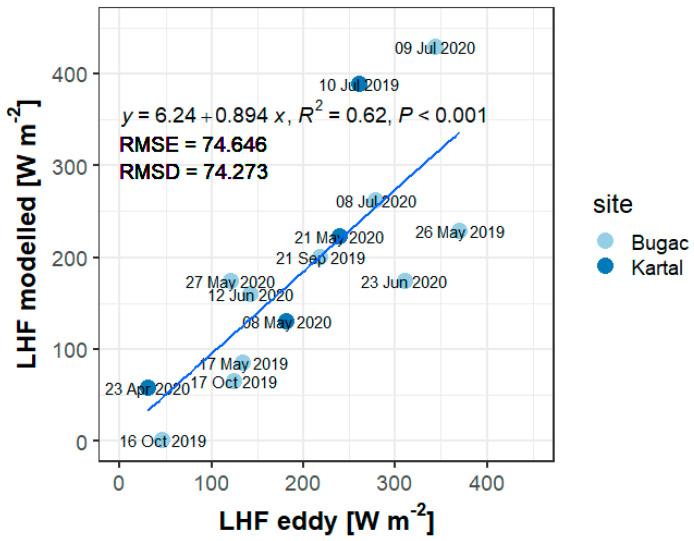
Comparison of modelled (pyTSEB based on thermal orthomosaic produced from UAV flight) and measured (eddy covariance) latent heat fluxes from a grassland (Bugac) and from a cropland (Kartal). UAV flights were performed during 2019–2020.

There is a statistically significant (*p* < 0.001) correlation between the modelled and measured fluxes (though the error is still at the order of 75 W/m^2^), showing the reliability of the image acquisitions and processing methods as well as the pyTSEB model (Figure 6). The lack of energy balance closure is a common and continuously discussed issue concerning the eddy covariance technique [30]. Since the pyTSEB model attributes the imbalance to the LHF by definition, the same approach was used to close the EC energy balance at fluxes used for validation in this study. This method was also used in previous similar studies, e.g., [3,12] and also suggested by [31].

## 4. Discussion

### 4.1. Prospects for Thermal UAV Studies

While relatively easy access to satellite ET maps is already provided, these maps still have an insufficient spatial resolution for use in studies evaluating small plot field-scale experiments, or in plant phenotyping works. For this reason, the expectations towards high spatial resolution and accuracy of evapotranspiration maps keep the interest in UAV solutions. Moreover, regarding other practical considerations, i.e., the availability of satellite-based time series might be lower in regions with high average cloud cover [32] such as Hungary, UAV based imagery has obvious advantage. Recent off-the-shelf solutions are also targeting higher thermal resolution imagery necessary in plant disease surveys in addition to the above-mentioned purposes. Opportunities for flexibility in UAV based thermal/RGB systems

For the above reasons, we have developed an integrated UAV based system including a high-resolution thermal imager, suitable to produce the necessary raster inputs to a TSEB model. Taking into account the trade-offs between the flexibility of such a system in terms of suitable constituents (cameras and GNSS receivers) and the efforts and resources used during development, we developed our application by relying on existing modules. These modules are of either hardware (such as a Raspberry Pi) or software type (e.g., the software development kit (SDK) provided to the thermal camera OPTRIS). The capabilities of the RPi microcomputer (in terms of different ports/interfaces, including, RS232, GPIO, Ethernet, general USB and WiFi) and the flexibility of the SDK proved to be highly adaptable in controlling the operation of the thermal and the RGB cameras and communicating with the onboard GNSS receiver. In addition, these features are a good base to configure the system as to include further component sensors.

The Emlid GNSS M+ receiver used as a rover onboard the UAV proved to be a troublesome item during the first part of the work. Later technical advancements published by the developers of the unit and the release of a new model contributed to the improved capabilities (booting up speed and later stability) of the system. The use of the hot-shoe adapter connecting directly the RGB camera (Sony A6000) to the GNSS rover increased geolocation accuracy. Comparing the solutions with the different Emlid Reach receivers, the bias of georeferencing decreased from about 1.5 m (M+) to 0.2–0.5 m (M2).

The current configuration of the system allows acquisition of 8 cm spatial resolution thermal imagery from a flight altitude of 60 m and the accuracy of the mosaicked RGB imagery is around 10 cm. In comparison, commonly available off-the-shelf combined thermal/multispectral solutions of Micasense offer 0.35 to 0.17 m thermal spatial resolution from the same height.

The application requires a few ground control measurements (leaf area index estimations using a ceptometer, for example), micrometeorological data (radiation, wind speed, air temperature, and humidity) in addition to the thermal imagery to run the pyTSEB model.

### 4.2. Validation of Modelled ET Data against Eddy Covariance Measured ET Fluxes

Modelled latent heat flux data have been validated against half-hourly averages of latent heat fluxes measured by eddy covariance (Figure 6) with RMSE of 75 Wm^−2^w/m^2^ (RMSD 74 Wm^−2^w/m^2^). In the last couple of years, the TSEB model applied to UAV based thermal orthomosaic was successfully validated against EC data in several studies for various ecosystems, e.g., grasslands [13,14], a tree-grass mixed ecosystem [3], an agricultural area [12], peach and nectarine orchards [20], olive orchard [11], and vineyard [10]. RMSD of modelled and measured (EC) was 40–50 Wm^−2^ for grasslands [13,14,18], while RMSE was 94 Wm^−2^ for an agricultural area [12].

The choice of the energy balance closure method affects the magnitude of uncertainty of the measured eddy fluxes. In the majority of recent studies validating UAV based energy fluxes against EC measured fluxes, the imbalance is fully added to the latent heat flux as suggested by [31]. This method was found to yield the best validation result out of the considered ones (closing the balance fully at the expense of the sensible heat flux, the latent heat flux or according to the Bowen ratio) [18]. This however cannot be considered as evidence in favor of closing the balance by increasing the latent heat flux, only. Yet, latent heat is calculated as the remainder in the two-source energy balance model after allowing for calculating sensible heat and ground heat fluxes. It is this analogy for which we used the approach of closing the energy imbalance also of the eddy covariance measurements solely by increasing the latent heat flux.

## 5. Conclusions

We have used a reasonable amount of engineering effort to build a Raspberry single-board computer driven imaging system. The system is consisting of a thermal and an RGB camera, equipped with a small GNSS receiver enabling the user to apply PPK. As with thermal images, the system has higher spatial resolution than the current off-the-shelf products making it suitable for acquitting imagery for, e.g., plant phenotyping (or for evaluation of small experimental plots) purposes. Moreover, the data acquired by this system is sufficient to run the two-source energy balance (TSEB) model and to construct a map of evapotranspiration of, e.g., a plantation or a crop field. The model is (slightly) underestimating the latent heat flux. To improve the model in this context, investigations considering the methods for the energy balance closure of the EC data are probably good candidates.

## Figures and Tables

**Table 1:** Summary of the studies addressing UAV based ET modelling. Type of vegetation, UAV, thermal, and RGB or multispectral camera, and the ET model used are included in the table. After the type of camera the ground resolution (m) is given in bracket.

Vegetation	UAV	Thermal Camera Type and Its Ground Resolution (m)	RGB/Multispectral Camera Type and Its Ground Resolution (m)	ET Model	Reference
vineyard	Cessna TU206	FLIR Therma CAM SC640 FLIR (0.38–0.66)	ImperX Bob-cat B8430 digital cameras (R, G, B, NIR) (0.05–0.1)	TSEB/DATTUTDUT	[10]
drip-irrigated olive orchard	helicopter based UAV Microkopter	EasIR-9, Wuhan Guide Infrared Co., Wuhan, China (0.06)	Mini MCA-6, Tetracam, Inc., Chatsworth, CA, USA (0.033)	remote sensing energy balance	[11]
barley	Quest UAV Q300	Optris PI 450 (0.13)	NA	TSEB-PT	[12]
grassland	MikroKopter OktoXL	Optris Pi 400 (0.05)	Samsung ES80	OSEB/TSEB	[13,14]
vineyard	Aggie Air	ICI/7640-P (0.6)	0.15	TSEB	[15]
soybean	DJI S1000+	FLIR VUE ProR (NA)	Parrot Sequoia (NA)	Not applied	[16]
vineyard	AggieAir	0.6	0.1	TSEB	[4]
spearmint, potato, alfalfa	ATI Agbot	TAU 2 460 FLIR (0.13)	Micasense RedEdge (0.07)	METRIC	[17]
grass, shrub, trees	Quan-tum Systems Trinity F90+	Micasense Altum (R, G, B, RedEdge, NIR: 0.04, TIR: 0.67)	Sony RX1 RIII (0.01)	TSEB DTD	[18]
almond orchard	DJI Matrice 210	Micasense Altum (R, G, B, RedEdge, NIR: 0.026–0.03, TIR: 0.41–0.475)	Zenmuse X4S	METRIC	[19]
peach and nectarine trees	DJI S1000	FLIR A65 (0.06)	MicaSense RedEdge (0.08)	HRMET	[20]
olive orchard	TAROT 1000 RC	FLIR TAU 2 640 (0.034–0.055)	NA	Clumped Model	[21]

**Table 2:** Optical properties of the digital and thermal cameras used in the integrated system considering flying altitude (e.g., for resolution) of 60 m.

Model	Resolution (Pixels)	FOV °	Focal Length (mm)	Ground Resolution (cm)
Canon Ixus	3864 × 5152	-	5	2
Sony a600	4000 × 6000	-	16	1.3
Optris PI 400	382 × 288	29 × 22	13	10
Oprtis PI 640	480 × 640	60 × 45	10.5	8

**Table 3:** List of flight missions: date and time interval in UTC at the grassland (site: Bugac) and the cropland (site: Kartal).

Grassland		Cropland	
Date	Time (UTC)	Date	Time (UTC)
17 May 2019	9:28–9:43	10 July 2019	8:57–9:28
26 May 2019	12:34–12:55		
21 September 2019	9:55–10:09		
16 October 2019	14:05–14:20		
17 October 2019	8:45–9:05		
27 May 2020	12:53–13:09	23 April 2020	15:47–16:03
12 June 2020	8:21–8:39	8 May 2020	6:43–7:00
23 June 2020	9:45–10:02	21 May 2020	8:07–8:25
8 July 2020	8:38–8:56		
9 July 2020	9:14–9:33		

**Table 4:** Distance (m) of the GCP positions saved from the orthomosaics and their measured positions. The avg and sd (selected) variables are calculated only from the GCP positions excluded from the mosaicking procedure.

Site:	Bugac	Bugac	Bugac	Bugac	Kartal	Kartal	Kartal
Date:	27 May 2020	12 June 2020	23 June 2020	8 July 2020	23 April 2020	8 May 2020	21 May 2020
GCP1	0.7552	0.3189	0.5953	0.2415	0.0694	0.0119	0.0724
GCP2	0.5600	0.1331	0.3282	0.0886	0.1292	0.0691	0.0601
GCP3	0.5132	0.1798	0.3379	0.0553	0.0401	0.0446	0.0441
GCP4	0.4974	0.2615	0.5061	0.0730	0.0699	0.1019	0.0900
GCP5	0.5210	0.3934	0.2507	0.1272	0.0757	0.0660	0.0585
GCP6	0.2559	0.1745	0.5029	1.1399	0.0716	0.0700	0.0623
GCP7	0.3208	0.1789		0.0238			
GCP8	0.2725	0.2100	0.6777	0.1686	0.0054	0.0735	0.0295
GCP9	0.0903	0.1206	0.5124	0.2040			0.0411
GCP10	0.4091	0.2420	0.4233	0.2981	0.0454	0.0113	0.0348
GCP11	0.5931	0.2224	0.3893	0.2841	0.1520	0.0643	0.0581
GCP12	0.6726	0.3761	0.2972	0.1554	0.0852	0.0645	0.0213
GCP13	0.5930	0.1435	0.2641	0.1629	0.0529	0.0591	0.0238
GCP14	0.6039	0.1799	0.3137	0.3151	0.0542	0.0514	0.0527
GCP15							0.0277
GCP16					0.0680	0.1102	0.0580
avg (selected)	0.5050	0.1811	0.3304	0.1315	0.0636	0.0904	0.0532
sd (selected)	0.1285	0.0323	0.0630	0.1179	0.0093	0.0274	0.0282

## Data Availability

Not applicable.
